# A bibliometric and visualization analysis of entosis research from 2007 to 2024

**DOI:** 10.3389/fonc.2024.1424100

**Published:** 2024-10-28

**Authors:** Xinyu Yang, Jiatao Tu, Xinyi Zang, Xuan Huang, Ye Tao

**Affiliations:** Department of Gastroenterology, The First Affiliated Hospital of Zhejiang Chinese Medical University (Zhejiang Provincial Hospital of Traditional Chinese Medicine), Hangzhou, China

**Keywords:** entosis, cell-in-cell, VOSviewer, CiteSpace, R package “bibliometrix”

## Abstract

**Objective:**

In 2007, entosis was proposed as a form of programmed cell death, distinct from apoptosis. This process involves a living cell (internalized cell) actively invading a neighboring live cell of the same type (host cell), forming a cell-in-cell structure. Recently, entosis has been increasingly associated with cancer, leading to significant advancements in research. Despite this progress, a comprehensive and unbiased review of the current state of entosis research is lacking. This study aims to evaluate the developments in the field of entosis over the past decade and highlight emerging research trends.

**Materials and methods:**

We performed a literature search for studies published since the introduction of the entosis concept, using the Web of Science Core Collection database. The bibliometric analysis was conducted using VOSviewer, CiteSpace, Microsoft Excel, and the Bibliometrix R package.

**Results:**

A total of 196 articles from 39 countries and 346 institutions were included. Between 2007 and 2024, research on entosis has seen rapid growth, with most publications originating from China and the United States. The United States also leads in total citations, with Memorial Sloan Kettering Cancer Center emerging as the top research institution. Sun Qiang is the most prolific author in this field, while Overholtzer M has the highest number of citations. *Current Molecular Medicine* has published the most articles related to entosis. Frequently occurring keywords include “entosis,” “cannibalism,” “autophagy,” and “apoptosis.” In recent years, keywords such as “phagocytosis,” “drug resistance,” and “human cancers” have surged, indicating a growing focus on understanding the role of entosis in tumor progression and exploring its potential as a therapeutic target for cancer treatment.

**Conclusions:**

This study provides the first bibliometric analysis of entosis, detailing its evolution over the last decade. It highlights critical areas of interest, including the development of inhibitors targeting entosis and their potential clinical applications. This research aims to guide future investigations and serve as a valuable resource for scholars exploring entosis in cancer biology.

## 1 Introduction

Entosis, proposed in 2007, refers to a form of programmed cell death distinct from apoptosis (1). This process involves a living cell (internalized cell) actively penetrating a neighboring live cell (host cell) of the same type, resulting in the formation of a cell-in-cell (CIC) structure. Several outcomes are possible for the cells involved in entosis. The most common fate for the internalized cell is death, mediated by the lysosomal pathway of the host cell. However, a small fraction of internalized cells can survive within the host, be re-released, and, in some cases, even undergo division and proliferation (2).

Initial studies on entosis suggested that it primarily results in cell death, which may provide anticancer benefits. For example, Overholtzer et al. (2007) found that entosis restricts the abnormal proliferation of tumor cells grown in soft agar, supporting its potential role in tumor inhibition (3). Additionally, the inhibition of entotic cell death through autophagy protein knockdown increases the transformed growth of cells with high rates of entosis, suggesting that entosis may reduce such growth, at least partially, by inducing cell death (4).

As research has progressed, however, the focus has shifted toward the idea that entosis may promote tumor cell proliferation and metastasis. Entosis induces aneuploidy, a known driver of tumor development. During entosis, the internalized cell can disrupt the host cell’s division by interfering with the cleavage furrow, often leading to cytokinesis failure (5, 6). This failure can result in binucleated cells with significant aneuploidy, contributing to the formation of melanoma lesions (7). Consistently, host cells can recover nutrients from digested entotic cells, a process known as nutrient scavenging, which helps support cell proliferation and survival under starvation conditions (8). With the growing understanding of entosis as a promoter of tumor progression, the development of entosis inhibitors has emerged as a promising area of research. For instance, a study published in *Cell Death & Disease* demonstrated that, in the absence of glucose, the inhibition of PEPCK-M with iPEPCK-2 promotes entosis, while overexpression of PEPCK-M inhibits it (9). Furthermore, recent studies have identified Ca^2+^ chelators and inhibitors of SEPTIN, Orai1, and MLCK as suppressors of entosis (10). The future discovery and development of additional entosis inhibitors hold great promise for their application in clinical anticancer treatments, potentially leading to advanced therapeutic approaches.

Entosis, one of the emerging modes of cell death, has garnered increasing attention from researchers in the field of cancer biology. The growing number of studies on entosis reflects a rapidly expanding interest in understanding its role, placing a significant demand on researchers to stay updated on the latest findings, track current research hotspots, and anticipate future developments in the field. Bibliometric analysis, a quantitative analytical method, employs mathematical and statistical tools to systematically examine the literature of a specific research domain. This method helps construct a knowledge network within a field, enabling researchers to identify key areas of study and the boundaries of academic discourse. The objectivity and rigor of bibliometric analysis have made it a widely used tool across numerous disciplines, allowing scholars to quickly understand prevailing trends in their respective fields (11, 12). Bibliometric analysis can more accurately assist researchers in the field of entosis to understand the latest scientific discoveries, identify research hotspots, and predict the future development of the field.

However, no bibliometric studies have yet been conducted on entosis. To address this gap, our study searched the Web of Science Core Collection for literature related to cellular entosis published from January 1, 2007, to February 15, 2024. Using tools such as CiteSpace, VOSviewer, and R (version 4.2.2), we performed a comprehensive bibliometric analysis and visualized the results. Our aim was to summarize the current state of entosis research, assess emerging trends, and predict future research directions in the field (13–15).

## 2 Materials and methods

### 2.1 Literature source and retrieval strategy

**Figure 1:**
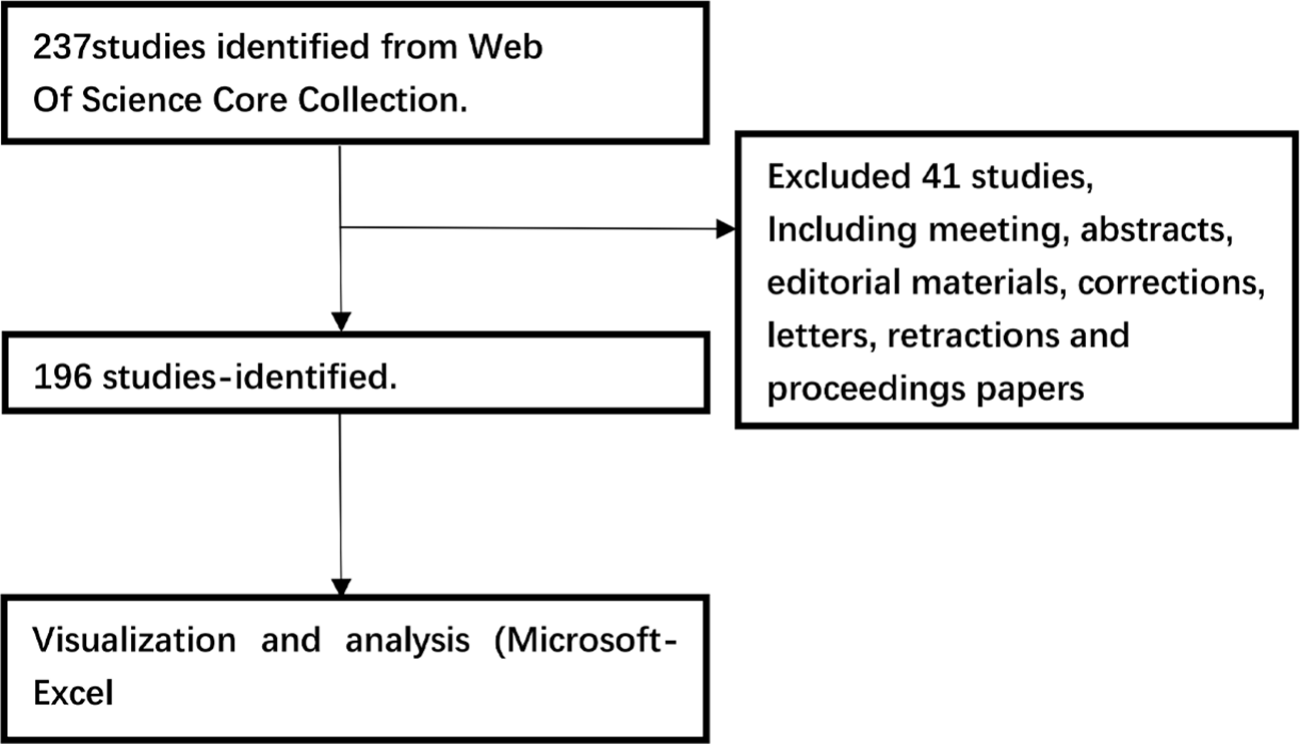
The flow chart depicting bibliometric analysis.

Data were retrieved from the Web of Science Core Collection on a single day, February 15, 2024, using the search formula TS = (entosis). The search covered literature published from January 1, 2007, to February 15, 2024. Out of the 237 articles initially identified, 41 were excluded, including meeting abstracts, editorial content, corrections, correspondences, retractions, and conference papers. Ultimately, 196 articles were selected for analysis and exported under the categories “Full Record and Cited References” and “Plain Text.” To ensure comprehensive examination, the exported files were renamed as “download_*.txt” to enable processing in CiteSpace. The retrieval and analysis process is illustrated in **Figure 1**.

### 2.2 Data analysis and visualization

To ensure the accuracy and consistency of the literature data, we utilized the “DEAN” data-cleaning process proposed by Pan Wei et al. for pre-processing and screening the data (16). Following this, we analyzed and visualized the literature using four software programs: Microsoft Excel 2021, CiteSpace, VOSviewer, and R (version 4.3.1). CiteSpace, developed by Chao-Mei Chen, is the most widely used tool for bibliometric analysis, and version 6.1.6 was used in this study for institutional collaboration analysis, journal cluster analysis, co-citation clustering and bursts, keyword timelines, and keyword bursts (17). VOSviewer, created by Nees Jan van Eck and colleagues, focuses on analyzing bibliometric knowledge graphs (18), and version 1.6.20 were used to visualize and analyze countries, journals, cited authors, co-cited authors, high-impact papers, and keywords. Additionally, the Bibliometrix R package (version 4.1.4) (https://www.bibliometrix.org) was used to display publication output by country (19), illustrate international collaboration, and present thematic trend analyses and a three-field plot. We also visualized the annual output of the top five institutions, journals, and the top 10 authors. Microsoft Excel 2021 was used to examine annual publication trends and conduct curve fitting.

## 3 Results

### 3.1 Annual quantitative distribution of publications

**Figure 2:**
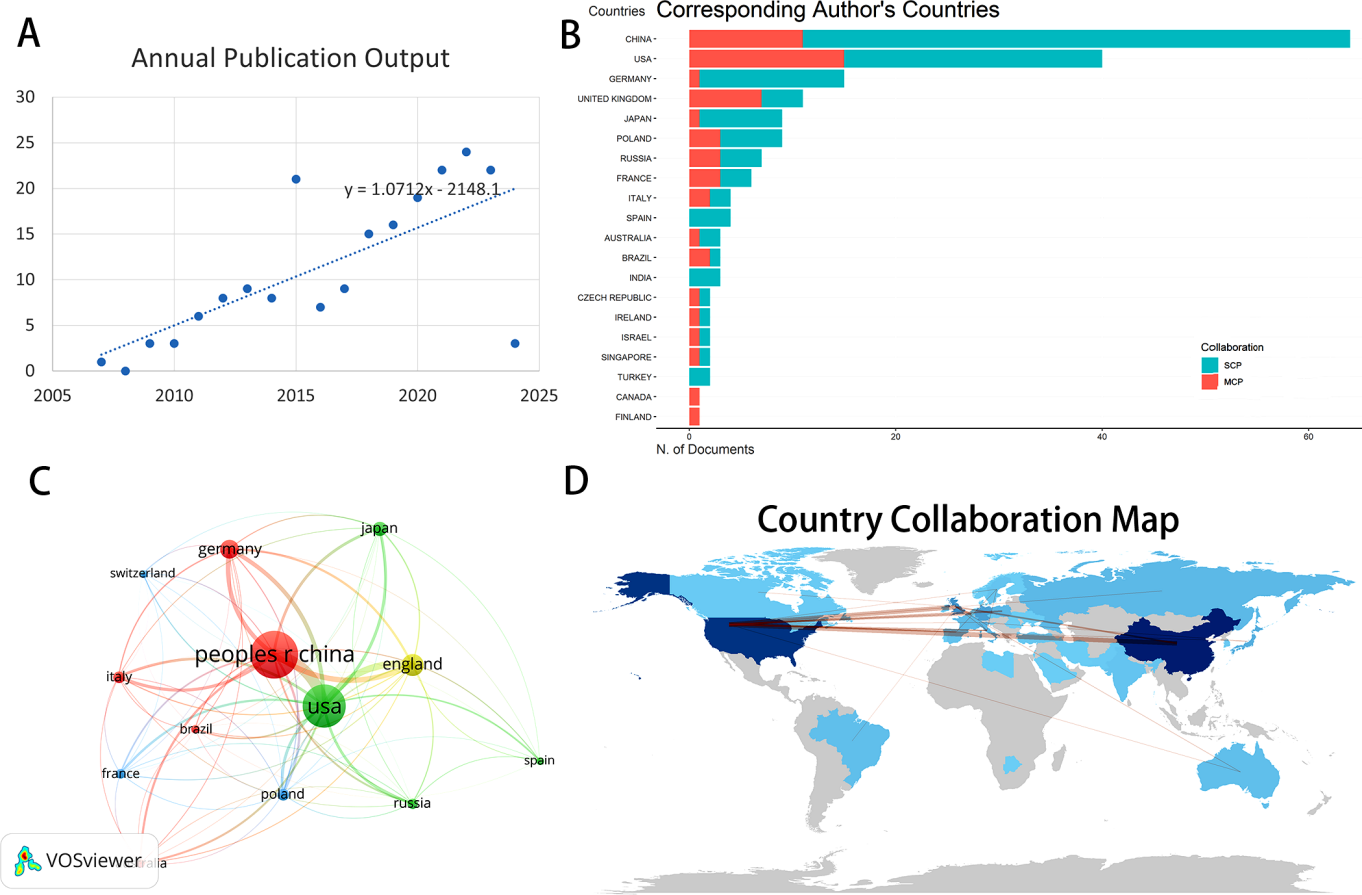
**(A)** Annual outputs of publications regarding Entosis field. **(B)** TOP 20 corresponding author’s countries that produced the largest number of literature. **(C)** The overlay visualization map of country co-authorship analysis conducted by VOSviewer. **(D)** The geographical network map of Entosis. SCP, Single Country Publications; MCP, Multiple Country Publications.

The annual publication of scholarly articles reflects the advancements in research within this domain. Our search strategy included all articles published since the definition of entosis was proposed, yielding a total of 237 English publications. After excluding publication types such as conference abstracts, book reviews, letters, and retracted articles, we conducted a comprehensive review of 196 papers, comprising 138 research articles and 58 reviews. Between 2007 and 2010, the number of publications was limited, indicating a period of stagnation in research, as illustrated in **Figure 2A**. However, following 2011, there has been a consistent increase in research literature in this field, with a peak in published articles observed from 2020 to 2023. This signifies a recent surge in recognition of entosis research among scholars. It is evident that entosis holds significant potential for clinical applications, particularly in anticancer therapy, and the trend in this research area is expected to continue expanding.

### 3.2 Analysis of countries and institutions

Since the introduction of the entosis definition, publications have emerged from 39 different countries and regions, as well as 346 organizations. As depicted in the geographical network map in **Figure 2D**, the top 10 countries were primarily located in Asia, Europe, and North America.

**Table 1** shows that China (71 publications, 36.22%) and the United States (61 publications, 31.12%) together accounted for 67.34% of the total global publications, significantly distancing themselves from the third-ranked country. The U.S. publications garnered the highest overall citation frequency (6,024), followed by the UK (3,230), France (2,808), and Italy (2,558). **Figure 2C** illustrates the frequency of international academic collaboration, with the size of each node representing the number of publications from that country. Collaborative research efforts among various nations were strong, with dynamic partnerships notably between the U.S., China, and the UK. Furthermore, Italy and the UK showed a strong partnership. However, China’s multi-country publication research constituted only a small fraction of its domestic research output (see **Figure 2B**), indicating a limited degree of close academic collaboration with other countries and continents in this research area. By incorporating these advanced analytical tools, we provide a comprehensive review of entosis-related research, establishing a solid foundation for assessing the current state of the field and its future potential.

**Table 1:** Top 10 nations and institutes for Entosis research.

Rank	Country	Citation	Articles	Institution	Year	Count (%)
1	China	1886	71(36.22%)	Memorial Sloan Kettering Cancer Center	2011	24(12.24%)
2	USA	6024	61(31.12%)	Capital Medical University	2014	19(9.69%)
3	England	3230	23(11.73%)	Chinese People's Liberation Army General.	2013	18(9.18%)
4	Germany	506	18(9.18%)	Cornell University	2011	16(8.16%)
5	Japan	316	12(6.12%)	Weill Cornell Medicine	2011	15(7.65%)
6	Italy	2558	9(4.59%)	Southern Medical University- China	2009	12(6.12%)
7	Poland	72	9(4.59%)	Chinese Academy of Sciences	2009	12(6.12%)
8	Russia	48	8(4.08%))	Biotechnology and Biological Sciences Res	2013	10(5.10%)
9	France	2808	7(3.57%)	South China University of Technology	2009	9(4.59%)
10	Australia	2515	6(3.06%)	Babraham Institute	2014	9(4.59%)

**Figure 3:**
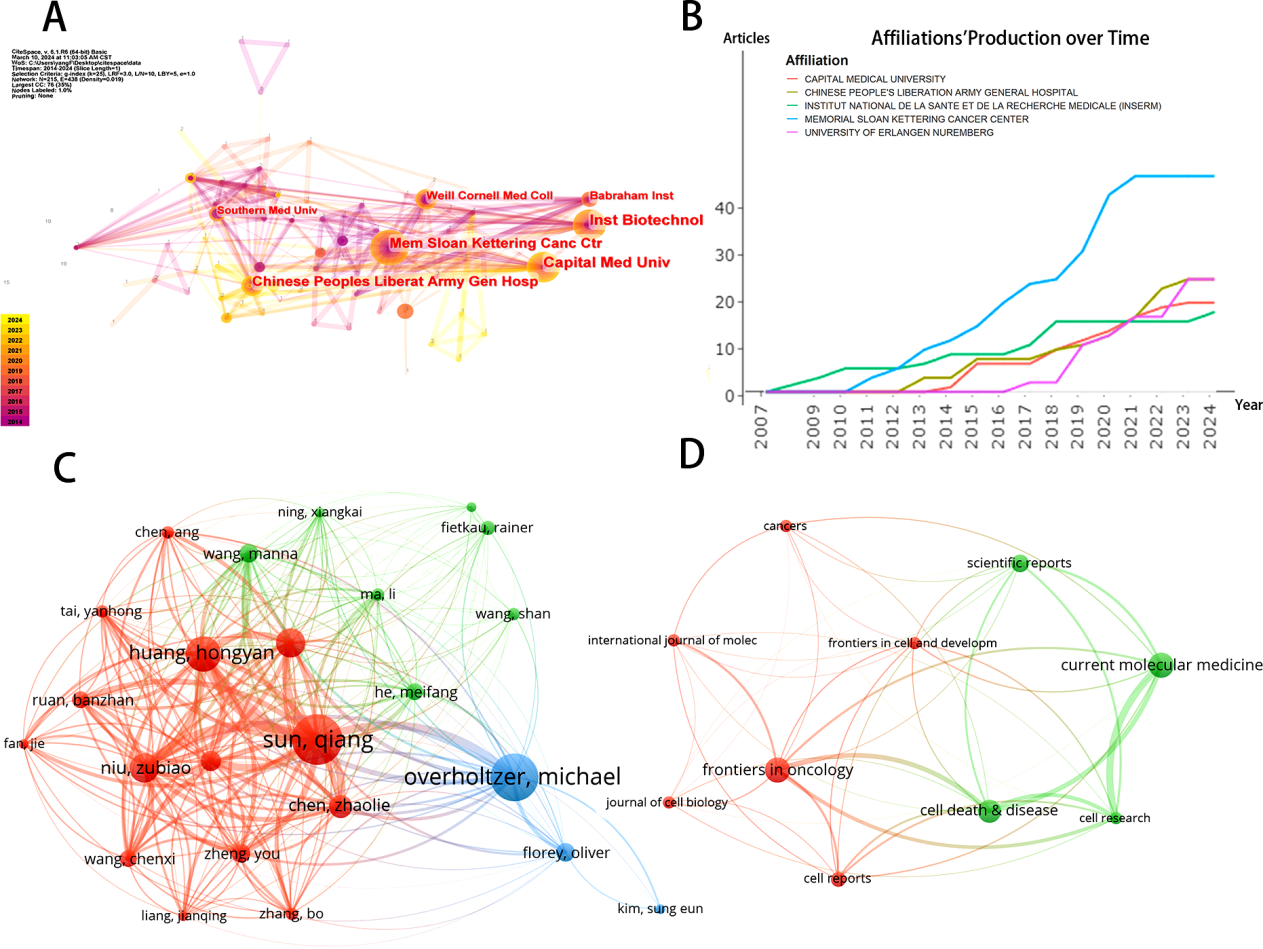
**(A)** The visualization of institutions' cooperation networks based on CiteSpace. **(B)** Top 5 institutions’ production over time. **(C)** Map of authors’ cooperative relationship. **(D)** Visualization of journals on the research of entosis based on VOSviewer.

Using CiteSpace software, we visualized and analyzed the 346 institutions involved in this research (**Figure 3A**). The ranking of publications is presented in **Table 1**, which highlights the top 10 institutions contributing to this field. The leading research institution is Memorial Sloan Kettering Cancer Center, with 24 publications, accounting for 12.24% of the total output. Notably, the list of institutions also includes several Chinese research entities. The diagram in **Figure 3A** shows the network of interinstitutional collaboration. As shown, Memorial Sloan Kettering Cancer Center actively collaborates with The Babraham Institute, while Capital Medical University maintains close ties with the Chinese People’s Liberation Army General Hospital. Moreover, partnerships among institutions are predominantly confined to their respective countries, reflecting a lack of dynamic academic cooperation across various nations. **Figure 3B** indicates a steady upward trend in the annual publication output of the top five institutions in recent years.

### 3.3 Journals and co-cited journals

**Figure 4:**
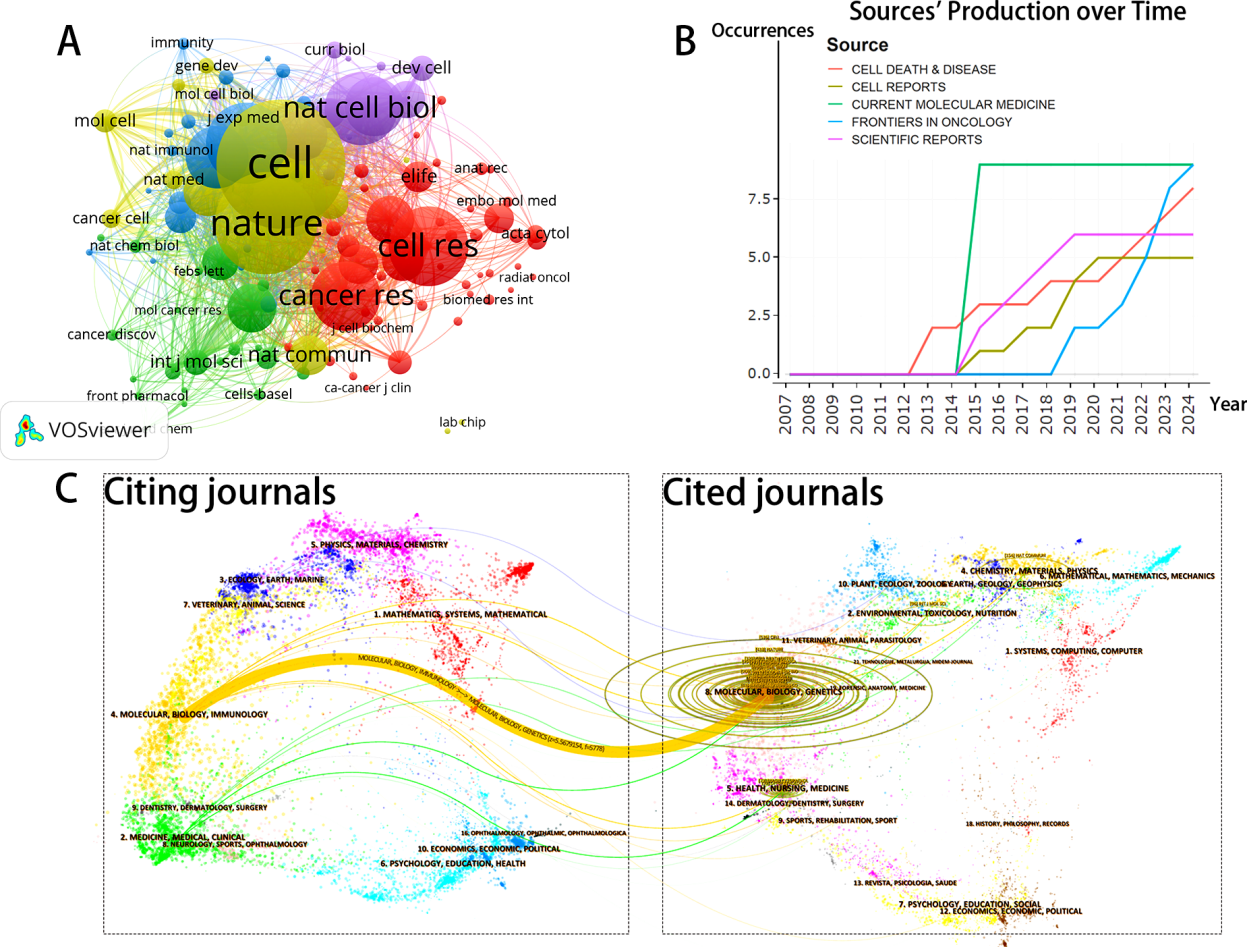
**(A)** Network map of journals that were co-cited in more than 20 citations. **(B)** Top 5 journals’ publication over time. **(C)** The dual-map overlay of journals related to Entosis.

The journals and co-cited journals within this research domain were visualized and analyzed using VOSviewer. A total of 119 journals were included in this analysis. **Figure 3D** displays the top 10 journals based on the number of publications, with a minimum threshold of 4 publications; the size of the nodes corresponds to the volume of publications for each journal. **Figure 4A** presents the network layout of journals cited together, including those with a minimum of 20 citations. This figure shows that the 119 journals cited together are represented in the overall strength of the links. Leading the list of journals in terms of overall link strength are *Cell* (total link strength = 38,061), *Nature* (total link strength = 35,478), *Cell Death & Differentiation* (total link strength = 26,500), *Proceedings of the National Academy of Sciences USA* (total link strength = 22,200), and *Journal of Biological Chemistry* (total link strength = 19,828). There has been a consistent increase in the publication numbers of the leading five journals in this field of study in recent years, as illustrated in **Figure 4B**.

**Table 2** numbers the top 10 most prolific and co-cited journals included in this study. *Molecular Medicine*, with an impact factor (IF) of 2.50 in 2024, emerged as the leading publisher with nine publications. Additionally, there were nine articles published in *Cell and Developmental Biology* (IF = 5.50, 2024), eight in *Cell Death & Disease* (IF = 9.00, 2024), and four in *Cell Research* (IF = 44.1, 2024). Notably, 7 out of the top 10 journals classified under JCR Region I contained relevant articles.

**Table 2:** Top 10 journals and co-cited journals for studies Entosis.

Ranks	Journals	Count	IF	Q	Co-Cited Journals	Co-Citation	IF	Q
1	*Current Molecular Medicine*	9	2.5	Q3	*Cell*	537	64.5	Q1
2	*Frontiers in Cell and Developmental Biology*	9	5.5	Q1	*Nature*	418	64.8	Q1
3	*Cell Death and Disease*	8	9	Q1	*Cell Death Differ*	328	12.4	Q1
4	*Scientific Reports*	6	4.6	Q2	*Cell Res*	322	44.1	Q1
5	*Cell Reports*	5	8.8	Q1	*P Natl Acad Sci USA*	296	11.1	Q1
6	*Cell Research*	4	44.1	Q1	*Nat Cell Biol*	292	21.3	Q1
7	*Frontiers in Cell and Developmental Biology*	4	5.5	Q1	*Cancer Res*	278	11.2	Q1
8	*International Journal of Molecular Sciences*	4	5.6	Q1	*J Biol Chem*	249	4.8	Q2
9	*Cancers*	4	5.2	Q2	*J Cell Biol*	235	7.8	Q1
10	*Journal of Cell Biology*	4	7.8	Q1	*Nat Rev Mol Cell Bio*	200	112.7	Q1

The journal biplot overlay provides a visual representation of the distribution of journals, the evolution of citation trajectories, and the shift in research focus across various research areas. In each subplot of **Figure 4C**, the left side displays the citing journal groups, while the right side illustrates the cited journal groups. As shown in **Figure 4C**, most citations in the curve are concentrated in the yellow citation link area, with the thickest citation link identified using the Z-score function of CiteSpace. The results indicate that the citing journals clustered around entosis primarily encompass molecular biology and immunology, whereas the cited journals cluster predominantly within the fields of molecular biology and genetics. These findings suggest that current research on entosis is mainly focused on molecular biology and immunology.

### 3.4 Authors and co-cited authors

**Figure 5:**
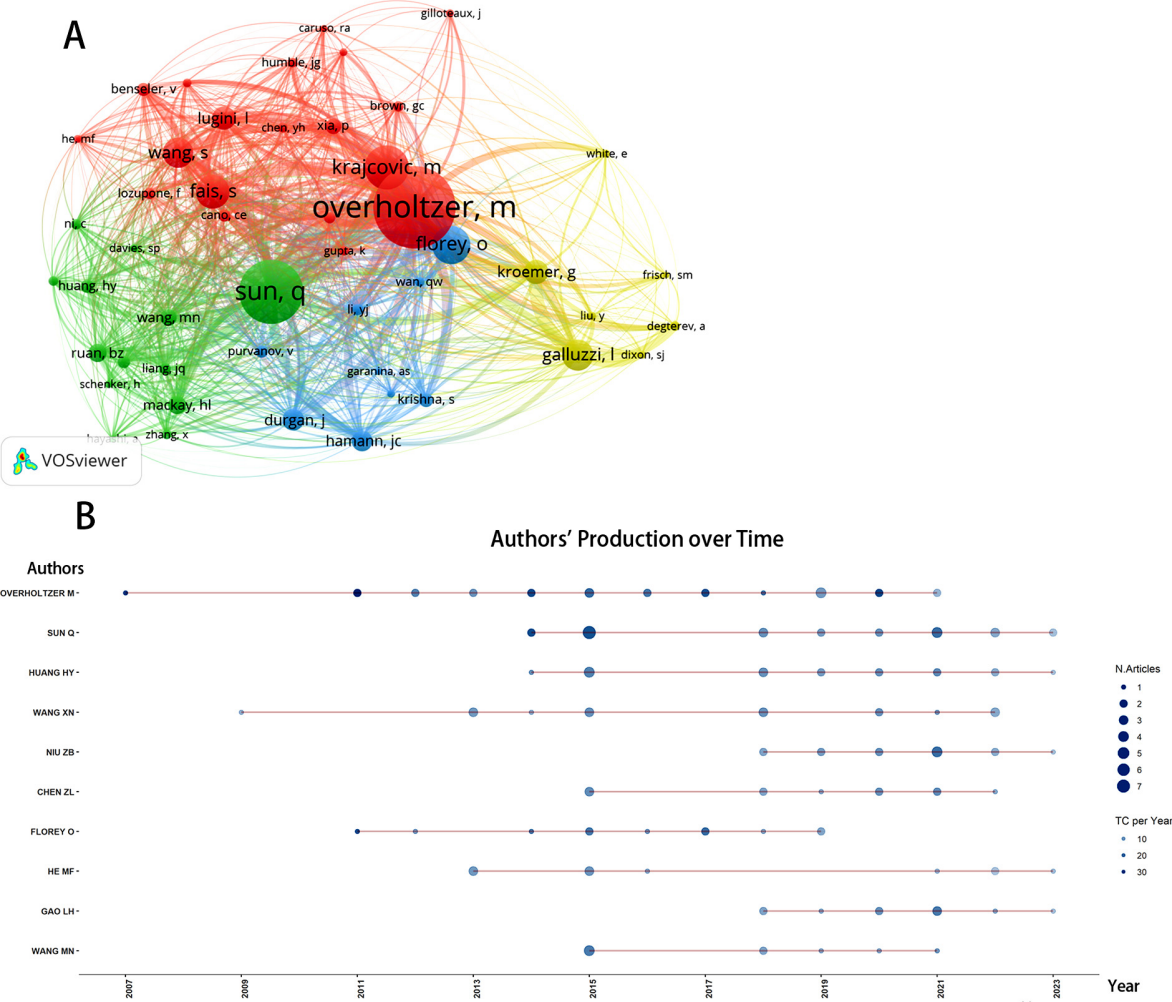
**(A)** Network visualization diagram of the co-cited authors regarding Entosis. **(B)** Top 10 authors’ production over time. TC, total citation.

A total of 1,055 authors have contributed to entosis research. As presented in **Table 3**, the top 10 authors contributed 140 publications, accounting for approximately 71% of all publications in this domain. Sun, Qiang emerged as the leading author with 24 research publications, followed by Overholtzer, M with 23 papers, and Huang, Hongyan with 17 papers. **Figure 5B** illustrates the yearly contributions of the leading 10 authors from 2007 to 2024. Overholtzer, M has been active in the field since the introduction of the concept of entosis, while Wang, Xiaoning and Florey, Oliver began their major research contributions after 2009 and 2011, respectively. As depicted in **Figure 3C**, VOSviewer illustrates the connections among authors, highlighting that those from the same countries or regions often collaborate more frequently and exhibit stronger connections. However, collaboration among authors from different countries remains inadequate.

**Table 3:** Top 10 authors and co-cited authors on Entosis.

Rank	Authors	Counts	Co-Cited Authors	Citations	Total Link Strength
1	Sun, Qiang	24	Overholtzer, M	240	22186
2	Overholtzer, M	23	Sun, Qiang	187	17261
3	Huang, Hongyan	17	Florey, O	129	12227
4	Niu, Zubiao	14	Fais, S	112	10369
5	Wang, Xiaoning	14	Galluzzi, L	99	9371
6	Chen, ZhaoLie	11	Wang, S	90	8695
7	Gao, Lihua	10	Kroemer, G	90	7401
8	Zheng, You	9	Lugini, L	71	6767
9	Wang, Manna	9	Durgan, J	546	612
10	Florey, O	9	Hamann, Jc	539	6066

### 3.5 Co-citation analysis

The co-citation analysis evaluates the relevance of scholarly articles based on their co-citation frequency. Using VOSviewer, we examined 48 authors who each have at least 20 citations. As illustrated in **Figure 5A**, notable collaborations include those between Qiang Sun and Manna Wang, as well as between Overholtzer, M and Fais, S. **Table 3** lists these authors, showing that Overholtzer, M is the most cited author (240 citations), followed closely by Sun, Q (187 citations) and Florey, O (129 citations). Notably, four authors in total have surpassed the 100-citation mark.

### 3.6 Highly valuable papers

**Figure 6:**
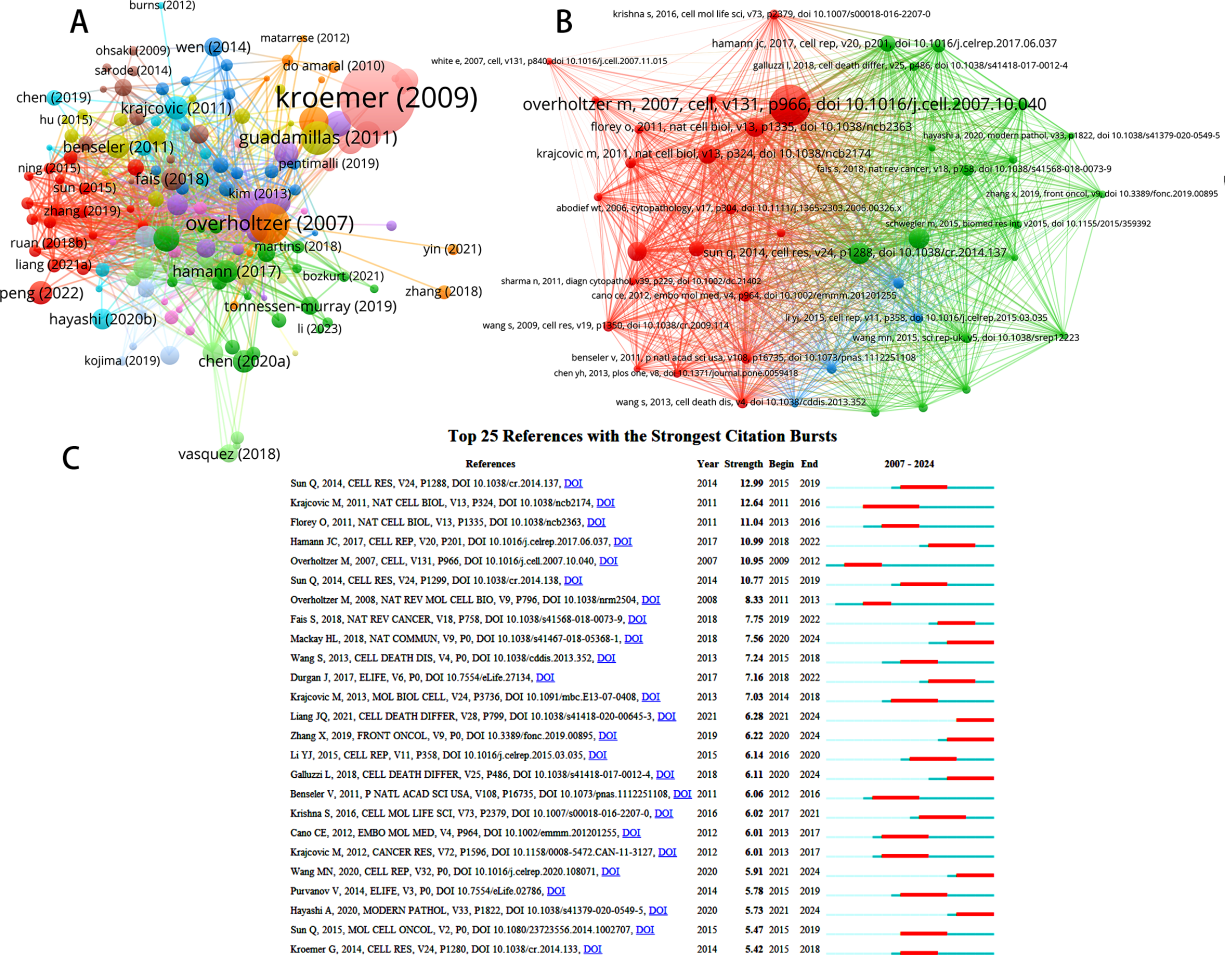
**(A)** Network map of citation analysis of documents with more than 5 citations. **(B)** Network map of co-citation analysis of references. **(C)** Top 25 references with strongest citation bursts of publications regarding Entosis.

To assess the impact of key papers on entosis studies, we analyzed citation counts from various regions. In total, over 150 papers in this area received more than five citations (**Figure 6A**). The paper titled “Classification of Cell Death: The 2009 Recommendations of the Committee on Nomenclature of Cell Death” has garnered an impressive 2,314 citations. This study details the NCCD’s 2009 recommendations regarding cell death terminology, including terms like “entosis,” “mitotic catastrophe,” “necrosis,” “necroptosis,” and “pyroptosis.” (20). It emphasizes that “entosis is a default pathway that becomes apparent only when other catabolic processes are inhibited.”

The second most cited article, “A Non-Apoptotic Cell Death Process, Entosis, That Occurs by Cell-in-Cell Invasion,” has received 499 citations. This paper introduces the concept of “entosis” and provides evidence of its role in the CIC cytological features commonly observed in human cancers (3). The third most cited article, “Autophagy Machinery Mediates Macroendocytic Processing and Entotic Cell Death by Targeting Single Membranes,” has been cited 332 times and discusses how autophagy proteins can target single-membrane vacuoles in cells even in the absence of pathogenic organisms (4).

VOSviewer identifies three primary groups (**Figure 6B**) with extensive interconnections among the references, highlighting the intricate relationships in this research area. **Table 4** presents the 10 most frequently cited sources in entosis research, with “Overholtzer M, 2007, Cell, v131, p966” standing out as the second most frequently cited reference, accumulating 169 citations.

**Table 4:** Top 10 co-cited references for Entosis research.

Ranks	Cited Reference	Citations	Total Link Strength
1	Overholtzer M, 2007, *Cell*, v131, p966,	169	1415
2	Sun Q, 2014, *Cell Res*, v24, p1288, doi 10.1038/cr.2014.137	78	955
3	Sun Q, 2014, *Cell Res*, v24, p1299,	75	981
4	Krajcovic M, 2011, *Nat Cell Biol*, v13, p324,	69	791
5	Florey O, 2011, *Nat Cell Biol*, v13, p1335,	68	777
6	Overholtzer M, 2008, *Nat Rev Mol Cell Bio*, v9, p796,	68	764
7	Hamann Jc, 2017, *Cell Rep*, v20, p201,	56	623
8	Lugini L, 2006, *Cancer Res*, v66, p3629,	53	703
9	Durgan J, 2017, *Elife*, v6,	42	523
10	Fais S, 2018, *Nat Rev Cancer*, v18, p758,	41	562

Additionally, references that have been widely cited by scholars in a specific field over time are referred to as references with citation bursts. These citations serve as important indicators, highlighting sources that have generated significant scholarly interest within a particular area over a specific period. Using CiteSpace, we identified the top 25 sources with the most intense citation surges, as shown in **Figure 6C**. Notably, Qiang Sun’s 2014 article, “Induction of Entosis by Epithelial Cadherin Expression” (21), ranked highest (intensity = 10.11). Following this, Krajcovic et al. defined a previously unknown mechanism of cytokinesis failure and aneuploid cell formation in human cancers (5), further exploring the role of entosis in this context.

### 3.7 Analysis of keywords

**Figure 7:**
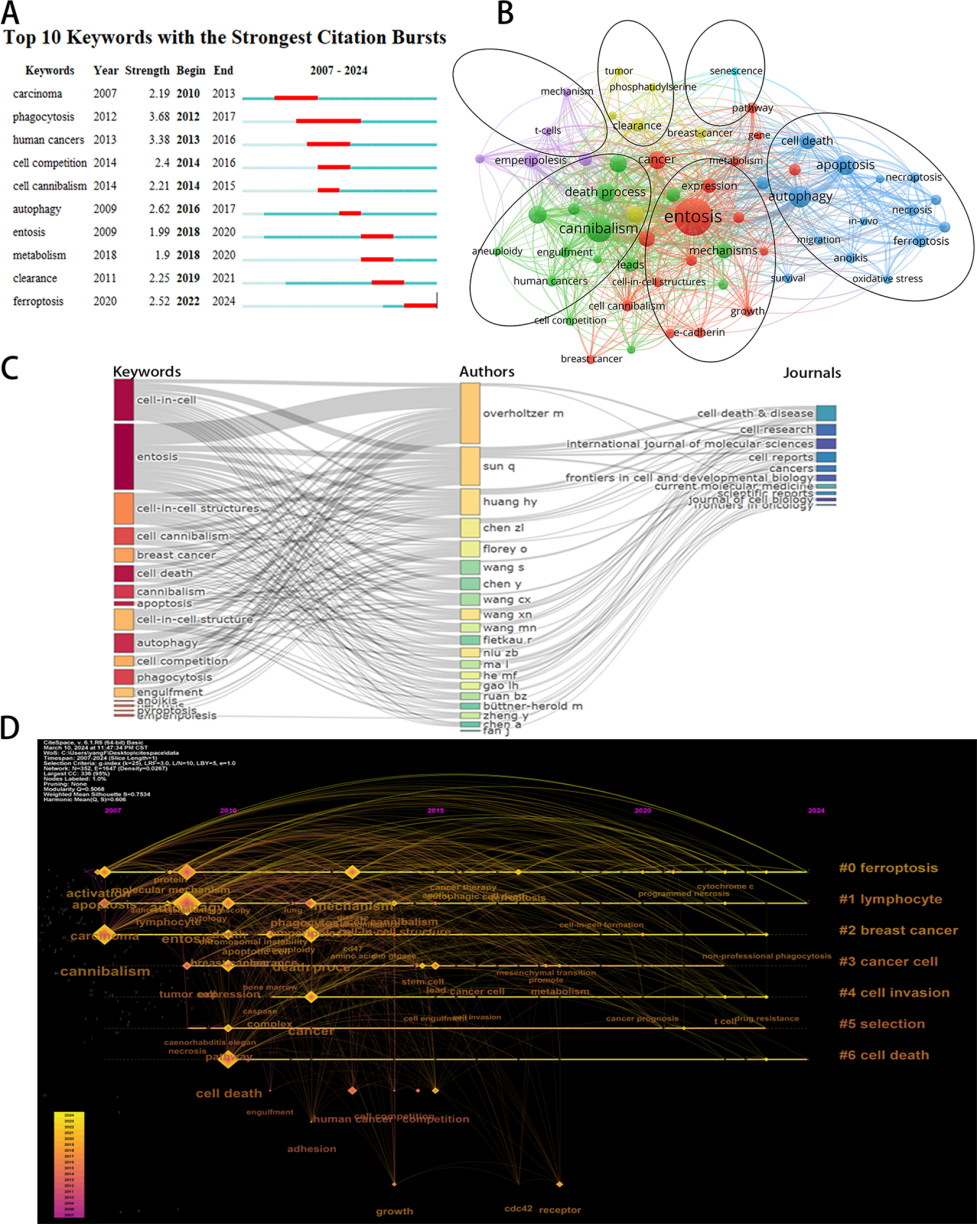
**(A)** Top 10 keywords with the strongest citation bursts based on Citespace. **(B)** Overlay visualization of the keywords network based on Vosviewer. **(C)** The three-field plot of the Keywords analysis base on R package “bibliometrix.” **(D)** The timeline view of keywords conducted by CiteSpace.

CiteSpace’s algorithm was used to detect keyword bursts. **Figure 7A** displays the top 10 keywords with the most significant bursts. The most frequently cited keyword was “phagocytosis” (intensity = 3.68), followed by “human cancers” (3.38) and “autophagy” (2.62). The keyword “phagocytosis” had the longest burst duration, spanning six years from 2012 to 2017. Notably, the keyword “ferroptosis” has seen a recent surge in citations (2022–2024). Ferroptosis is a novel iron-dependent mode of programmed cell death that differs from apoptosis, necrosis, and autophagy. It is closely linked to the pathophysiological processes of various diseases, including tumors and neurological disorders (22). Given that entosis and ferroptosis are both forms of programmed cell death, their simultaneous appearance in studies related to tumor cellular mechanisms suggests that research into entosis’s role in tumor development may become a significant area of interest in the future.

Analyzing keyword co-occurrences facilitates the rapid identification of hotspots within the research domain. The leading 20 high-frequency terms in this area are enumerated in **Table 5**. According to the co-occurrence analysis, the top four keywords identified were: “entosis” (137 occurrences), “cannibalism” (58 occurrences), “autophagy” (42 occurrences), and “apoptosis” (35 occurrences). Cannibalism, autophagy, and apoptosis were mentioned more than 30 times and represent the primary focus of entosis research.

**Table 5:** Top 20 keywords on research of Entosis.

Rank	Keywords	Occurrences	Rank	Keywords	Occurrences
1	Entosis	137	11	Death	21
2	Cannibalism	58	12	Mechanisms	19
3	Autophagy	42	13	Emperipolesis	18
4	Apoptosis	35	14	Leads	16
5	Cell-in-cell	28	15	Expression	16
6	Cancer	28	16	Tumor-cells	15
7	Death process	27	17	Lymphocytes	15
8	Phagocytosis	26	18	Ferroptosis	14
9	Carcinoma	24	19	Activation	14
10	Cell death	23	20	Clearance	13

From a total of 1,111 keywords, we excluded those with fewer than five occurrences and selected 59 keywords for cluster analysis using VOSviewer. As illustrated in **Figure 7B**, the keywords were categorized into six distinct clusters, each representing a unique research pathway. The red cluster includes “entosis,” “expression,” and “gene,” indicating a strong correlation between entosis and the selective expression of genes. The blue cluster encompasses “autophagy,” “apoptosis,” and “cell death,” suggesting a potential connection between entosis and various forms of cell death, particularly apoptosis. The purple cluster, characterized by terms like “emperipolesis,” “lymphocytes,” and “nurse cells,” indicates a higher incidence of entosis in specific cell types, such as lymphocytes. The green cluster features keywords like “cannibalism,” “engulfment,” and “death process,” emphasizing the concept of cannibalism among similar cells in entosis. Lastly, the orange cluster contains keywords such as “breast cancer,” “clearance,” and “tumor,” highlighting that entosis has been extensively studied in solid tumors, particularly breast cancer.

**Figure 7C** presents a three-field graph linking authors, keywords, and journals, showcasing the most frequently used keywords and the journals that publish the most in this field. The most common keywords include “CIC,” “entosis,” “CIC structures,” and “cell cannibalism.” Authors Sun Q, Overholtzer M, and Huang HY are closely associated with the keywords “entosis” and “CIC,” creating strong links. The journal with the most significant connections is *Cell Death & Disease*.

Analyzing the timeline of keywords provides valuable insights into the progress and evolving focus of the research area. The size of the nodes reflects the frequency of keyword appearances within the clusters, with earlier nodes representing keywords that appeared first in the timeline. **Figure 7D** depicts keywords such as “ferroptosis,” “lymphocyte,” “breast cancer,” “cancer cell,” “cell invasion,” “selection,” and “cell death.” The early appearance of “cannibalism” (dating back to 2007) suggests that cancer and apoptosis were among the initial areas of interest in this field. Notably, the frequency of the keyword “drug resistance” has significantly increased recently. This trend may indicate the rapid development of entosis within cancer therapeutics, suggesting that the development of novel drugs aimed at countering cancer cell escape is becoming a prominent research topic.

## 4 Discussion

This investigation used tools such as VOSviewer, CiteSpace, and the Bibliometrix R package to implement sophisticated bibliometric analysis principles and advanced visualization techniques. Our review of entosis and its impact on cancer involved an extensive analysis of annual publications, geographical regions, institutions, contributing and cited authors, academic and peer-reviewed journals, and pertinent keywords, aiming to uncover major research foci and trends in this domain.

In examining geographical areas and institutions, China and the United States emerged as the leading countries in terms of publication output. However, there is a notable lack of international collaboration between these countries. To foster the collective advancement of this field, China must actively engage in strengthening global cooperation. Chinese institutions account for the highest percentage of the top 10 institutions by publication volume. While the development of entosis research requires the exchange and collaboration of experts, these institutions predominantly collaborate with domestic counterparts. We anticipate an increase in cooperation among different countries and institutions to promote collaborative growth in this research area.

Regarding journals and co-citation analysis, *Current Molecular Medicine* ranked first in terms of publication count. In 2024, the top 10 journals had an average IF of 9.86, with *Cell Research* recording the highest IF at 44.10. Notably, 80% of the journals had an IF exceeding 5, and 70% of these journals were categorized as Q1 (JCR). Among the journals cited together, *Nature Reviews Molecular Cell Biology* emerged as the highest in IF at 112.70, while *Cell* was the most frequently cited. Furthermore, 9 out of the top 10 journals frequently cited were classified as Q1. These results indicate that this research area has attracted significant academic attention from prominent journals specializing in cell biology and cancer treatments.

In terms of authorship, Sun, Qiang, and Overholtzer, Michael stand out as the most prolific contributors in the field. The collaboration network among authors (**Figure 3C**) highlights their ongoing cooperative efforts. Their most cited article, “Competition between Human Cells by Entosis,” demonstrates that human cells engage in direct competition through a phagocytic process known as entosis, resulting in their engulfment or cannibalism while alive, followed by cell death. Our research reveals that the characteristics of the engulfing (“winner”) and engulfed (“loser”) cells are determined by the phagocytic mechanism governed by RhoA and actomyosin. In heterogeneous populations, tumor cells with high deformability tend to engulf and outcompete neighboring cells with lower deformability. Furthermore, the study found that the activation of the Kras and Rac pathways bestows dominant status on cells by reducing contractile myosin, facilitating the absorption of adjacent cells, which ultimately leads to cell death. The paper also calculates the energy dynamics of CIC formation, highlighting the mechanical distinctions between winning and losing cells as crucial for advancing our understanding of entosis. This information outlines a competitive process in mammalian cells, particularly in human tumors, and serves as a vital guide for examining entosis in human cancer cells.

Within the context of cited authors, Overholtzer, M emerges as the most frequently cited author. He is best known for his article that first introduced the concept of “entosis,” providing evidence that it is a prevalent “CIC” phenomenon in human cancers. This foundational article not only established the concept of entosis but also suggested that it is driven by the compaction forces associated with the formation of adherens junctions, occurring without integrin interaction. This mechanism may potentially serve as an inherent tumor-inhibiting force for cells separated from the extracellular matrix.

The significance of the most cited literature and references in this field has been discussed in detail above; therefore, these points will not be reiterated here.

### 4.1 Hotspots and frontiers

Recent research has highlighted that references and keywords experiencing a surge in citations can serve as indicators of trending topics within a specific field. Upon examining the major research areas associated with these frequently cited references and keywords, we identified that current themes in entosis research primarily focus on understanding the biological mechanisms underlying entosis and exploring the potential of inducing entosis in cancer cells as a therapeutic strategy. Notably, references citing the surge in 2014 reported that the expression of exogenous epithelial cadherin proteins (E- or P-cadherin) in human breast tumor cells, which lack endogenous expression of these cadherins, induces entosis and inhibits transformed growth (21).

Keywords are an effective means of quickly grasping the focus and progress within the entosis research field. By employing both keyword clustering analysis and a timeline perspective (see **Figure 7**), we can discern temporal shifts in research topics. Current trends increasingly concentrate on the potential impact of entosis on tumor cells and the application of entosis to impede cancer progression. This focus reflects a strong interest in the practical implications of entosis research, particularly in uncovering the effects of entosis on the development of tumor and entosis inhibitors. Thus, we can conclude that research on entosis has focused on the following areas:

### 4.2 Hotspots: impact of entosis on tumor development

Entosis is characterized by the dynamic penetration of a living cell (the internalized cell) into an adjacent living cell of a similar type (the host cell), leading to the formation of a CIC structure. While entotic cells typically undergo death, some can escape from the host cell. A small percentage of these cells are capable of division within the host (3). The death of entotic cells occurs through a non-cell-autonomous process, as the live engulfed cells are degraded by autophagy and lysosomal pathways (23). This cell death occurs in the absence of cysteine-3 cleavage and lacks the morphological features associated with apoptosis. Therefore, it has been suggested that entosis should be classified as a novel type IV cell death (24).

Initial research indicated that CIC structures had an anticancer effect primarily by inducing cell death (3, 25). Early experiments demonstrated that the entosis process could inhibit tumor progression by killing tumor cells that had already been isolated from the stroma (5). However, entosis may promote tumor progression by inducing changes in cell ploidy (6). It actively fosters polyploidy through the interruption of cell division, leading to the development of polyploid cells in cultures. Furthermore, there is a correlation between CIC structures and cancer stages; for instance, in lung (26), gastric (27, 28), breast (29), renal (30), and pancreatic cancers (31), CIC structures serve as markers of poor prognosis. A direct comparison of xenograft growth in mice revealed that cells exhibiting greater endodermal activity formed larger tumors, indirectly hinting at a pro-tumorigenic role for the CIC structure (32). As research on entosis has advanced, evidence has accumulated that a significant proportion of cancer cells undergoing entosis are able to escape from host cells (32, 33). It is now widely accepted that host cells provide a safe environment for endocytes, allowing them to evade adverse conditions such as nutrient deprivation, toxic substances, and immune cell attacks (34, 35). In conclusion, entosis is increasingly viewed as promoting the proliferation and metastasis of tumor cells.

### 4.3 Hotspots: inhibitors of entosis

Entosis is triggered by various physiological conditions, including matrix detachment, aberrant mitosis (36), and glucose deprivation (37), Regardless of the initiating mechanism, entotic cells form adherens junctions (AJs) through Ca^2+^/E-cadherin interactions (21). Following the formation of AJs, these cells are engulfed via actin polymerization (38), mechanical ring formation (39), and actomyosin contraction (40–42).

Recent findings indicate that intracellular Ca^2+^ signaling regulates entosis through the SEPTIN-Orai1-Ca^2+^/CaM-MLCK-actomyosin axis (40, 41). Intracellular Ca^2+^ oscillations in entotic cells exhibit spatiotemporal variations during engulfment, mediated by Orai1 Ca^2+^ channels in the plasma membrane (43). SEPTIN controls the polarized distribution of Orai1, leading to local MLCK activation, which results in MLC phosphorylation and actomyosin contraction, ultimately facilitating the internalization of invasive cells (44–46). Notably, Ca^2+^ chelators and inhibitors of SEPTIN, Orai1, and MLCK have been shown to suppress entosis (10).

As the role of entosis in tumor development becomes clearer, investment in research and development of entosis inhibitors is expected to grow, paving the way for future anticancer therapies that target this process.

## 5 Conclusion

In summary, a bibliometric analysis of entosis research reveals a rapidly evolving and dynamic field that reflects our growing understanding of novel modes of cell death and the potential for anticancer therapies to make a significant impact. Key themes identified include “cancer prognosis,” “drug resistance,” and “cell engulfment.” As research in entosis has advanced, there is a noticeable shift toward the perspective that entosis promotes tumor cell proliferation and metastasis, underscoring the current emphasis on the entotic properties of tumor cells. Notably, the emergence of “entosis inhibitors” as a central topic suggests a promising direction for future research, as the pro-cancer effects of entosis continue to be elucidated. The integration of cellular pharmacology, cell biology, and related disciplines may provide a robust impetus for the development of entosis inhibitors. For instance, the identification of Orai1 as a Ca^2+^ channel involved in non-apoptotic cell death and its role in cancer development has facilitated the development of Ca^2+^ chelators, as well as inhibitors targeting SEPTIN, Orai1, and MLCK to inhibit entosis. Consequently, researchers, clinicians, and policymakers should closely monitor this field and support its ongoing advancement, given its significant potential to inhibit tumor cell growth of tumor cells.

## Data Availability

The original contributions presented in the study are included in the article/supplementary material. Further inquiries can be directed to the corresponding author.
